# Diagnostic Yield and Complication Rate of Stereotactic Biopsies in Precision Medicine of Gliomas

**DOI:** 10.3389/fneur.2022.822362

**Published:** 2022-03-30

**Authors:** Sophie Katzendobler, Anna Do, Jonathan Weller, Mario M. Dorostkar, Nathalie L. Albert, Robert Forbrig, Maximilian Niyazi, Rupert Egensperger, Niklas Thon, Joerg Christian Tonn, Stefanie Quach

**Affiliations:** ^1^Department of Neurosurgery, University Hospital, LMU Munich, Munich, Germany; ^2^Center for Neuropathology and Prion Research, LMU Munich, Munich, Germany; ^3^Department of Nuclear Medicine, University Hospital, LMU Munich, Munich, Germany; ^4^German Cancer Consortium (DKTK), Partner Site Munich, German Cancer Research Center (DKFZ), Heidelberg, Germany; ^5^Institute of Neuroradiology, University Hospital, LMU Munich, Munich, Germany; ^6^Department of Radiation Oncology, University Hospital, LMU Munich, Munich, Germany

**Keywords:** stereotactic biopsy, glioma, recurrent glioma, pseudoprogression, precision medicine, molecular diagnostics, image-guided procedures

## Abstract

**Background:**

An integrated diagnosis consisting of histology and molecular markers is the basis of the current WHO classification system of gliomas. In patients with suspected newly diagnosed or recurrent glioma, stereotactic biopsy is an alternative in cases in which microsurgical resection is deemed to not be safely feasible or indicated. In this retrospective study, we aimed to analyze both the diagnostic yield and the safety of a standardized biopsy technique.

**Material and Methods:**

The institutional database was screened for frame-based biopsy procedures (January 2016 until March 2021). Only patients with a suspected diagnosis of glioma based on imaging were included. All tumors were classified according to the current WHO grading system. The clinical parameters, procedural complications, histology, and molecular signature of the tissues obtained were assessed.

**Results:**

Between January 2016 and March 2021, 1,214 patients underwent a stereotactic biopsy: 617 (50.8%) for a newly diagnosed lesion and 597 (49.2%) for a suspected recurrence. The median age was 56.9 years (range 5 months−94.4 years). Magnetic resonance imaging (MRI)-guidance was used in 99.3% of cases and additional positron emission tomography (PET)-guidance in 34.3% of cases. In total, stereotactic serial biopsy provided an integrated diagnosis in 96.3% of all procedures. The most frequent diagnoses were isocitrate dehydrogenase (IDH) wildtype glioblastoma (*n* = 596; 49.2%), oligodendroglioma grade 2 (*n* = 109; 9%), astrocytoma grade 3 (*n* = 108; 8.9%), oligodendroglioma grade 3 (*n* = 76; 6.3%), and astrocytoma grade 2 (*n* = 66; 5.4%). A detailed determination was successful for IDH 1/2 mutation in 99.4% of cases, for 1p/19q codeletion in 97.4% of cases, for TERT mutation in 98.9% of cases, and for *MGMT* promoter methylation in 99.1% of cases. Next-generation sequencing was evaluable in 64/67 (95.5%) of cases and DNA methylome analysis in 41/44 (93.2%) of cases. Thirteen (1.1%) cases showed glial tumors that could not be further specified. Seventy-three tumors were different non-glioma entities, e.g., of infectious or inflammatory nature. Seventy-five out of 597 suspected recurrences turned out to be post-therapeutic changes only. The rate of post-procedural complications with clinical symptoms of the Common Terminology Criteria for Adverse Events (CTCAE) grade 3 or higher was 1.2% in overall patients and 2.6% in the subgroup of brainstem biopsies. There was no fatal outcome in the entire series.

**Conclusion:**

Image-guided stereotactic serial biopsy enables obtaining reliable histopathological and molecular diagnoses with a very low complication rate even in tumors with critical localization. Thus, in patients not undergoing microsurgical resection, this is a valuable tool for precision medicine of patients with glioma.

## Introduction

Gliomas represent a heterogeneous group of neoplasms of the central nervous system. Classification and subsequent management decisions depend on histological and molecular features. The WHO provides the framework for classification which leads to the guidelines for clinical management (1–5).

Hence, both histology and molecular diagnosis are mandatory in newly diagnosed intracerebral lesions suspicious for glioma. This can be obtained either by tumor resection or stereotactic biopsy. Whether the patient should undergo an open, microsurgical tumor resection or just a biopsy depends mainly on the clinical status of the patient, location and extent of the lesion, and the patients' preference. Gross total resection is associated with better long-term outcome but also inherits a risk of perioperative and postoperative complications despite modern neurosurgical techniques (6–8). Conversely, biopsies are not used for the reduction of tumor volume and but are administered for tissue-based diagnosis only (9). They can be minimally invasive, provide both histological and molecular diagnosis, and may be more suitable for multimorbid or frail patients with very high surgical risk factors for midline tumors or patients with gliomas in highly eloquent areas of the brain bearing a high functional risk in case of extensive tumor reduction.

Especially in *MGMT* methylated glioblastomas, and also in IDH mutated gliomas, treatment-induced changes on conventional magnetic resonance imaging (MRI) are not always easily distinguishable from true tumor progression, a phenomenon termed pseudoprogression (10, 11). Despite the added value of advanced MRI including MR perfusion and MR spectroscopy and positron emission tomography (PET) using radiolabeled amino acids (e.g., O-(2-^18^Ffluorethyl)-L-tyrosine ([^18^F]FET PET)) to assess the real tumor burden (12–14), tissue sampling provides the gold standard of information for further management of these uncertain cases.

Tumor relapse is not only a hallmark of IDH wild type glioblastoma but also occurs frequently in lower grade, IDH mutant gliomas (15–17). Patients, thus, are often subjected to a multitude of therapies over time given the fact that, so far, no standard treatment for recurrent gliomas exists. Individualized, targeted therapy is an emerging field in the treatment of gliomas and tissue sampling is necessary to identify the druggable targets using next-generation sequencing. Drugs directed against receptor tyrosine kinases (RTK) and downstream molecules like PI3K/AKT/mTOR as well as drugs targeting the mitogen-activated protein kinase (MAPK) signaling pathway are currently under investigation (2, 18, 19). Small-molecule inhibitors targeting IDH mutations are being tested in clinical trials (NCT02073994, NCT02481154). As mutational landscapes of gliomas may change during therapy and disease course, a safe and efficient way to obtain glioma tissue for identification of targetable molecular alterations would be of great benefit (20).

Thus, there is a growing need to obtain a tissue-based diagnosis even at multiple points in time during the clinical course of glioma. A minimally invasive approach would be desirable to accomplish the goal of having maximally informative specimens with minimal risk and burden for the patient. Whether risks and gains of stereotactic biopsies are well-balanced has been a matter of debate for a long time (21). However, the diagnostic yield in the framework of a molecular-driven brain tumor diagnosis and the associated complication rates of biopsies initially and during clinical course have not yet been investigated comprehensively. In this retrospective study, we aimed at analyzing both the diagnostic yield and the safety of a standardized biopsy technique between 2016 and 2021 in a single high-volume center with a high number of tertiary referrals.

## Materials and Methods

### Patient Evaluation

The local database of the Department of Neurosurgery of the University Hospital Munich (Ludwig-Maximilians University) was screened for all biopsy procedures in a 5-year period between January 2016 and March 2021. Only patients with a suspected diagnosis of glioma were included. After histological confirmation of a glioma through biopsy, molecular analyses were performed. Clinical parameters such as age at diagnosis, Karnofsky Performance Status (KPS), initial symptoms, date of stereotactic biopsy, postoperative clinical course, and last follow-up were assessed retrospectively. All patients or caregivers gave written informed consent. The local ethics committee of the University Hospital Munich approved the study (project number 325-2011).

### Biopsy Technique

**Figure 1:**
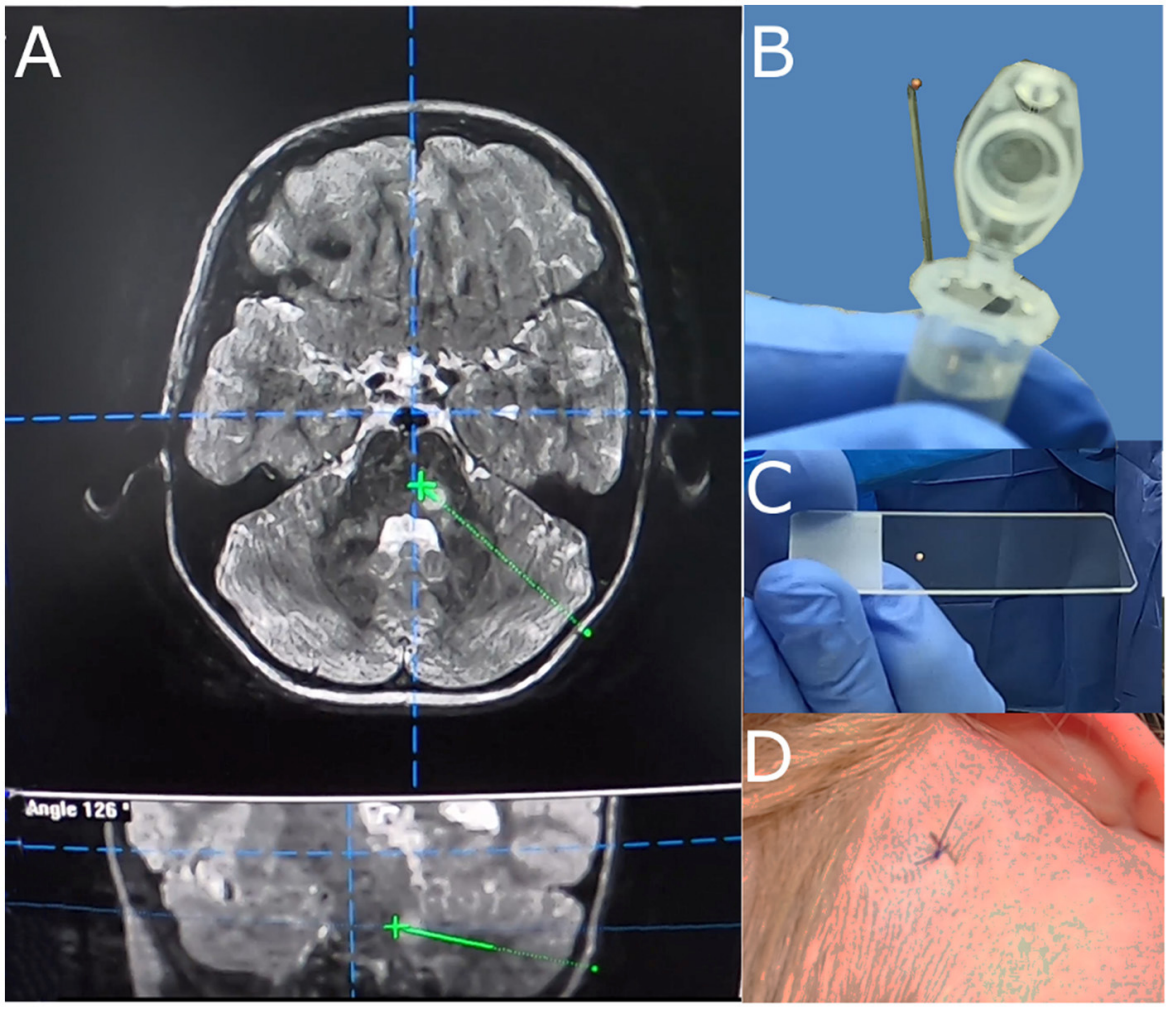
Biopsy trajectory planning **(A)**, sample size of acquired specimen [arrows, **(B,C)**], and skin incision **(D)**.

**Figure 2:**
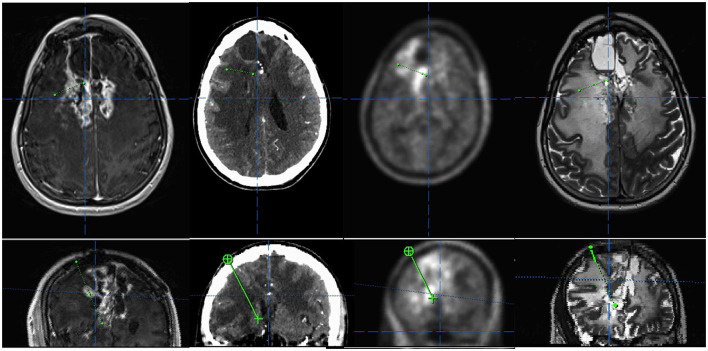
Example of a multimodal trajectory planning targeting both contrast- and (fluorethyl)-L-tyrosine (FET)-enhancing areas in a case of a suspected recurrence of a multimodally treated oligodendroglioma, IDH mutated and 1p/19q co-deleted, the central nervous system (CNS) WHO grade 3. **(Upper panel)** Axial view of contrast enhanced T1, CT, FET positron emission tomography (PET), and T2. **(Lower panel)** Inline view depicting the trajectory plane.

A standardized frame-based imaging-guided stereotactic biopsy technique was used in all patients. The preoperative workup comprised a 1.5 or 3T MRI scan (with T2 and T1 sequences before and after application of a Gadolinium-based contrast agent and MR-angiography sequences) that was acquired 1 day prior to surgery and fused with an intraoperative, contrast-enhanced CT angiography scan (Figure 1). If available, the PET imaging data based on [^18^F]FET PET was included in the triplanar trajectory planning (Figure 2). Each trajectory was meticulously planned to avoid any risk of vascular damage, contact to sulci, or drainage of the cerebrospinal fluid (CSF), which may lead to an intraoperative brain shift with a subsequent mismatch between planning MRI and real anatomy. A phantom frame was used to confirm the correct 3-dimensional angulation prior to the surgery in all patients. If present, the T1 contrast-enhancing lesions and/or suspicious [^18^F]FET PET foci were targeted. After attaching the frame under sterile conditions, a skin incision of 4–6 mm is made and followed by a frame-guided burr hole trepanation with a diameter of 3 mm. After perforation of the dura through advancing a sharp trocar, a blunt trocar inside a guiding tube (1.4 mm guide tube and trocar, Medical High Tech GmbH, Bad-Krozingen-Biengen, Germany) is used to reach the lesion. Subsequently, with the guide tube in place, multiple small tissue samples of 1 mm^3^ each are taken by utilizing the designated biopsy forceps (Medical High Tech GmbH, Bad-Krozingen-Biengen, Germany) inserted into the guide tube. Usually, 5–30 individual specimens per trajectory were taken depending on tumor size and the relation between solid tumor and necrosis. Thereafter, the skin is closed with a single stitch. The average length of the procedure, including the intraoperative CT scan, is 50.4 min.

An experienced neuropathologist is on site in the OR during the procedure to check *via* smear preparation whether the material obtained is sufficient in terms of quantity and quality for diagnosis.

### Complications and Follow-Up

Complications were classified according to the Common Terminology Criteria for Adverse Events (CTCAE 5.0; Supplementary Table 1) (22). Complications receding within 3 months were classified as transient, else they were classified as permanent. The routine follow-up after biopsy consisted of a postoperative CT scan on the first day after the procedure and an MRI follow-up in 3–6 months intervals for high-grade gliomas and low-grade gliomas, respectively.

### Histology and Molecular Markers

All glioma specimens were classified according to the WHO 2016 at the Center for Neuropathology and Prion Research of the University Hospital Munich and retrospectively re-classified according to the WHO 2021 (3). Routine molecular analysis at first diagnosis comprised immunohistochemical staining against R132H-mutated IDH1 and ATRX and PCR-based analysis of the IDH1 and 2 mutational hotspots, R312 and R172, respectively (PyroMark Q24 System, Pyro Gold reagents kit, Qiagen, Hilden, Germany); a microsatellite marker analysis was used for the detection of 1p and 19q deletions (23, 24). The mutations within the TERT promoter sequence were detected by the Sanger sequencing utilizing the QIAquick PCR Purification Kit (Qiagen, Hilden, Germany), the BigDye Terminator V3.1 Cycle Sequencing kit (Life Technologies, Carlsbad, USA), the DyeEX 2.0 Spin Kit (Qiagen, Hilden, Germany), and 3130 Genetic Analyzer (Life Technologies, Carlsbad, USA) (25). The DNA methylation status of the *MGMT* promoter was determined by bisulfite modification and subsequent nested methylation-specific PCR and sequencing analysis. Tumors were classified binarily as methylated or unmethylated (26). Further molecular analyses were initiated when the results were inconclusive or when aiming at identifying targetable mutations in patients with conventional treatment failure. In these cases, next-generation sequencing was performed using a combined DNA and RNA panel (Trusight Oncology 500, Illumina, San Diego, CA, USA). The DNA methylation profiling was performed for tumor not classifiable by other means or to detect clinically or diagnostically relevant copy number alterations such as homozygous CDNK2A/B deletions. The methylation profiling was done using an Illumina Infinium MethylationEPIC BeadChip array (Illumina, San Diego, CA, USA) with subsequent data analysis using the DNA methylation-based brain tumor classifier provided by the Deutsche Krebsforschungszentrum (v11b4) (27).

### Statistics

The final database contained patient-related, clinical, and tumor-specific information such as patient age at diagnosis, gender, clinical status utilizing the KPS, localization of the tumor, histological and molecular glioma features, and postinterventional complication rates. Based on this data, descriptive statistical analyses were performed utilizing the SPSS Statistics 25 software (IBM, Armonk, New York, USA).

## Results

### Patients and Procedural and Tumor Characteristics

In total, 1,214 consecutive biopsy procedures were analyzed. The median age of patients was 56.9 years (range 5 months−94.4 years). Of the total patients, 58.6% were men and 41.4% were women. A KPS of 80 or higher was reported in 82.1% of all patients. In 50.8% of cases, a biopsy was performed to obtain tissue in a newly diagnosed tumor and in 49.2% of cases for suspected recurrence. Image guidance was based on MRI in 99.3% cases and on CT in 0.7% cases due to contraindications for MRI imaging. Additionally, [^18^F]FET PET was used in 34.3% cases.

A total of 596 tumors (49.1%) were located on the left and 535 (44.1%) on the right side, and 83 patients (6.8%) had a bilateral midline tumor. The tumor site was lobar in 1,011 (83.3%), deep seated (insula, thalamus, corpus callosum, pineal region) in 123 (10.1%), cerebellar in 40 (3.3%), and brainstem in 40 (3.3%) patients (for detailed location see Table 1).

**Table 1:** Biopsy location in primary and recurrent diseases.

**Location**		**First diagnosis *n* (%)**	**Recurrence *n* (%)**	**Total *n* (%)**
Lobar	Frontal	155 (12.8)	232 (18.4)	378 (31.1)
	Temporal	158 (13.0)	161 (13.3)	319 (26.3)
	Parietal	79 (6.5)	78 (6.4)	157 (12.9)
	Occipital	15 (1.2)	12 (1.0)	27 (2.2)
	Pre-/postcentral gyrus	67 (5.5)	63 (5.2)	130 (10.7)
Deep-seated	Callosal	12 (1.0)	3 (0.2)	15 (1.2)
	Insular	27 (2.2)	26 (2.1)	53 (4.4)
	Thalamic	31 (2.6)	6 (0.5)	37 (3.0)
	Pineal	15 (1.2)	3 (0.2)	18 (1.5)
	Cerebellar	25 (2.1)	15 (1.2)	40 (3.3)
Brainstem	Mesencephalon	8 (0.7)	3 (0.2)	11 (0.9)
	Pons	14 (1.2)	4 (0.3)	18 (1.5)
	Medulla oblongata	11 (0.9)	0 (0.0)	11 (0.9)
Total		617 (50.8)	597 (49.2)	1,214 (100.0)

The most common diagnosis was glioblastoma IDH wild type with 596 cases (49.2%), followed by oligodendroglioma grade 2 (*n* = 109; 9.2%), astrocytoma grade 3 (*n* = 108; 8.9%), oligodendroglioma grade 3 (*n* = 76; 6.4%), astrocytoma grade 2 (*n* = 66; 5.4%), IDH 1/2 mutated astrocytoma WHO grade 4 (*n* = 45; 3.7%), and diffuse midline glioma, H3K27M- or FGFR1-mutated (*n* = 15+1; 1.3%) (Table 2).

**Table 2:** Histological diagnoses.

**Entity**		**Newly diagnosed lesion n (%)**	**Recurrence *n* (%)**	**Total *n* (%)**
Glioma	Glioblastoma, IDH wild type	354 (29.2)	243 (20.1)	596 (49.2)
	Midline glioma, H3K27M-mutated	12 (1.0)	3 (0.2)	15 (1.2)
	Astrocytoma WHO grade 4, IDH-mutant	4 (0.3)	41 (3.4)	45 (3.7)
	Astrocytoma WHO grade 3, IDH-mutant	19 (1.6)	89 (7.3)	108 (8.9)
	Astrocytoma WHO grade 2, IDH-mutant	34 (2.8)	32 (2.6)	66 (5.4)
	High-grade astrocytoma with piloid features	3 (0.2)	1 (0.1)	4 (0.3)
	Oligodendroglioma WHO grade 3, IDH-mutant and 1p/19q-codeleted	8 (0.7)	68 (5.6)	76 (6.3)
	Oligodendroglioma WHO grade 2, IDH-mutant and 1p/19q-codeleted	37 (3.0)	72 (5.9)	109 (9.0)
	Ganglioglioma	7 (0.6)	4 (0.3)	11 (0.9)
	Pilocytic astrocytoma	11 (0.9)	13 (1.1)	24 (2.0)
	Pleiomorphic xanthoastrocytoma	0 (0.0)	1 (0.1)	1 (0.1)
	Pleiomorphic astroglial tumor	2 (0.2)	0 (0.0)	2 (0.2)
	Ependymoma	1 (0.1)	1 (0.1)	2 (0.2)
	Anaplastic ependymoma	2 (0.2)	3 (0.2)	5 (0.4)
Other gliomas, not elsewhere classified (NEC)	*Glioma (NEC)*	1 (0.1)	0 (0.0)	1 (0.1)
	Glial tumor	11 (0.9)	1 (0.1)	12 (1.0)
	Glioneural tumor	7 (0.6)	0 (0.0)	7 (0.6)
	Neuroepithelial tumor	7 (0.6)	1 (0.1)	8 (0.7)
Other	Initially suspected glioma, diagnosis other than glioma	41 (3.4)	4 (0.3)	45 (3.7)
	Metastasis	31 (2.6)	2 (0.2)	33 (2.7)
	Medulloblastoma	3 (0.2)	4 (0.3)	7 (0.6)
	Meningioma	6 (0.5)	4 (0.3)	10 (0.8)
	Neurocytoma	3 (0.2)	2 (0.2)	5 (0.4)
	Germinoma	6 (0.5)	1 (0.1)	7 (0.6)
	Other entities (pineocytoma, neurinoma, diffuse leptomeningeal glioneuronal tumor, papillary tumor of the pineal region, pineoblastoma, solitary fibrous tumor, craniopharyngioma, yolk sac tumor)	7 (0.7)	7 (0.7)	14 (1.3)
Total		617 (50.8)	597 (49.2)	1,214 (100.0)

### Diagnostic Yield and Molecular Analyses

Among all newly diagnosed lesions, histopathology and molecular analyses provided a definite diagnosis in 595/617 cases (96.4%). Among the 22 unclear results, 14 patients were followed up by MRI imaging, as a low-grade tumor in an eloquent location was histologically and clinically the most likely diagnosis. None of these patients experienced tumor progression during a mean follow-up of 21 months. In six cases, the treatment was initiated based on recommendations by our interdisciplinary tumor board according to the most likely diagnosis (3 glial tumors without further subclassification; 3 diagnoses other than glioma). In only two cases, a second invasive procedure was required for obtaining the diagnosis: one patient underwent re-biopsy after 2 weeks, confirming IDH wild type glioblastoma, and another patient underwent open tumor resection revealing ganglioglioma.

Among all suspected recurrences, vital tumor was detected in 522 out of 597 cases (87.1%), while predominantly post-therapeutic changes were found in 75 cases (12.6%). In 3 cases (4% of all tissues showing post-therapeutic changes), recurrence within 3 months suggested a false negative sampling. In three cases with histologically diagnosed tumor recurrence (0.6%), further clinical course suggested mainly post-therapeutic changes, i.e., false-positive sampling. This amounts to a positive predictive value of 99.4% and a negative predictive value of 96%.

The standard molecular analyses, required by the WHO 2021 grading system, were successfully obtained in the vast majority of tumors being identified as gliomas by histology. The molecular status was informative for IDH 1/2 mutation in 99.4%, for 1p/19q codeletion in 97.4%, for TERT mutation in 98.9%, and for *MGMT* promoter methylation in 99.1%. Next-generation sequencing was attempted in 67 cases and evaluable in 64. The DNA methylation analysis was attempted in 44 cases and evaluable in 42. Twelve, thereof, showed no match with known methylation classes. Altogether, a successful molecular characterization for integrated diagnosis was obtained in 93% of all newly diagnosed and in 88.3% of all recurrent lesions.

### Complications

The routine postoperative CT showed no visible conspicuity in 816 (67.2%) cases, a minimal (<5 mm) hemorrhage in 305 (25.1%) cases, a local (>5 mm) hemorrhage in 51 (4.2%) cases, and a space-occupying hemorrhage in 10 (0.8%) cases. In 30 cases, no postoperative CT scan was performed in young patients without relevant deficit. Table 3 lists clinical complications in relation to imaging features. No clinical sequelae of the stereotactic biopsy were observed in 1,164 (95.9%) of procedures. Mild complications (CTCAE grade 1) were documented in 14 (1.2%) and moderate (CTCAE° 2) in 21 (1.7%) cases. Complications of CTCAE grade 3 occurred in 11 procedures (5 hemiparesis, 4 seizure series, 3 cases of delirium, 1 reduced level of consciousness, total 0.9%). Four patients (0.3%) required urgent intervention (CTCAE grade 4): three patients with postoperative bleeding required craniotomy and hematoma evacuation. One of these patients re-bled a second time after an initially successful hematoma evacuation and needed a second revision craniotomy, possibly due to a decreased level of fibrin stabilizing factor (factor XIII) diagnosed after the second revision surgery. All three patients with hematoma evacuation improved to CTCAE grade 1 or 0 within 3 months. One superficial wound infection required local debridement. Regarding the subgroup of brainstem lesions, two patients (5.3%) experienced mild complications and one (2.6%) a moderate complication (local hemorrhage with transient aggravation of a preexisting hemiparesis). In total, 74% of all clinical complications were resolved within 3 months (Table 4). There were no procedure-related deaths in the overall cohort.

**Table 3:** Complications according to postoperative imaging and severity.

**Blood on postoperative CT scan (*n*, % of total)**	**Clinical complications (CTCAE grade)**	**Newly diagnosed lesions; *n* (%)**	**Recurrent lesions; *n* (%)**	**Total; *n* (%)**
No visible blood (*n* = 816; 67.2%)	0 (none) 1 (mild) 2 (moderate) 3 (severe)	395 (98.0) 2 (0.5) 6 (1.5) 0	406 (98.3) 3 (0.7) 2 (0.5) 2 (0.5)	801 (98.2) 5 (0.6) 8 (1.0) 2 (0.2)
Minimal (<5 mm) hemorrhage (*n* = 305; 25.1%)	0 (none) 1 (mild) 2 (moderate) 3 (severe)	149 (96.1) 0 5 (3.2) 1 (0.6)	142 (94.7) 4 (2.7) 4 (2.7) 0	291 (95.4) 4 (1.3) 9 (3.0) 1 (0.3)
Local (>5 mm) Hemorrhage (*n* = 51; 4.2%)	0 (none) 1 (mild) 2 (moderate) 3 (severe) 4 (life-threatening)	26 (81.3) 2 (6.3) 1 (3.1) 2 (6.3) 1 (3.1)	16 (84.2) 1 1 (5.3) 1 (5.3) 0	42 (82.4) 3 (5.9) 2 (3.9) 3 (5.9) 1 (2.0)
Space occupying hemorrhage (*n* =10;0.8%)	2 (moderate) 3 (severe) 4 (life-threatening)	1 (16.7) 2 (33.3) 3 (50.0)	1 (25.0) 3 (75.0) 0	2 (20.0) 5 (50.0) 3 (30.0)
Ischemia (*n* = 2;0.8%)	0 (none) 1 (mild)	0 0	1 (50.0) 1 (50.0)	1 (50.0) 1 (50.0)
No imaging available (*n* = 30; 2.5%)	0 (none) 1 (mild)	20 (95.2) 1 (4.8)	9 (100) 0	29 (96.7) 1 (3.3)
Total (*n* = 1,214; 100%)	0 (none) 1 (mild) 2 (moderate) 3 (severe) 4 (life-threatening)	590 (95.6) 5 (0.8) 13 (2.1) 5 (0.8) 4 (0.6)	574 (96.1) 9 (1.5) 8 (1.3) 6 (1.0) 0	1,164 (95.9) 14 (1.2) 21 (1.7) 11 (0.9) 4 (0.3)

**Table 4:** Fraction of transient or permanent complications among all complications.

**Clinical complications (CTCAE grade)**	**Transient *n* (% of total)**	**Permanent *n* (% of total)**	**Total *n* (% total)**
1	12 (0.9)	2 (0.2)	14 (1.2)^a^
2	17 (1.4)	4 (0.3)	21 (1.7)
3	4 (0.3)	7 (0.6)	11 (0.9)
4	4 (0.3)	0 (0.0)	4 (0.3)
Total	37 (3.0)	13 (1.1)	50 (4.1)

a *Percentages do not add up due to rounding*.

### Brainstem Biopsies

A subgroup of 40 patients underwent a stereotactic biopsy of a brainstem lesion, whereof 13 were pediatric patients. The most frequent diagnosis was diffuse midline glioma, H3K27M mutated (*n* = 8), glioblastoma IDH wild type (*n* = 5), IDH 1/2 mutated astrocytoma (*n* = 7), and pilocytic astrocytoma (*n* = 5). All diagnoses of brainstem tumors are detailed in Table 5. In six cases, another diagnosis other than tumor was made, which was confirmed also by a further clinical course. NGS and DNA methylation analysis was attempted and successfully performed in three cases each. Two patients (5.3%) experienced mild complication and one (2.6%) patient had a moderate complication (local hemorrhage which transient aggravation of a preexisting hemiparesis).

**Table 5:** Diagnoses of brainstem biopsies in adult and pediatric patients.

	**Adult *n* (%)**	**Pediatric *n* (%)**	**Total *n* (%)**
Midline glioma	3 (11.1)	5 (38.5)	8 (20.0)
Glioblastoma, IDH wildtype	3 (11.1)	2 (15.4)	5 (12.5)
Astrocytoma, IDH mutated	6 (22.2)	1 (7.7)	7 (17.5)
Astrocytoma with piloid features	1 (3.7)	0 (0.0)	1 (2.5)
Oligodendroglioma, IDH mutated, 1p/19q codeleted	1 (3.7)	0 (0.0)	1 (2.5)
Pilocytic astrocytoma	3 (11.1)	2 (15.4)	5 (12.5)
Glial tumor, NEC	3 (11.1)	0 (0.0)	3 (7.5)
Glioneuronal tumor	1 (3.7)	0 (0.0)	1 (2.5)
Papillary tumor of the pineal region	0 (0.0)	1 (7.7)	1 (2.5)
Metastasis	2 (7.4)	0 (0.0)	2 (5.0)
Other diagnoses than tumor	4 (14.8)	2 (15.4)	6 (15.0)
Total	27 (100)	13 (100)	40 (100)

## Discussion

With the help of image-guided stereotactic biopsy, we could establish a histopathological and molecular diagnosis and distinguish true progression from pseudoprogression in a consecutive series of 1,214 patients with suspected glioma with a very high diagnostic accuracy of 96.4% in terms of histology, over 97% for molecular markers, and over 95% in 850 k/NGS arrays. The rate of non-gliomas among all suspected gliomas was low, possibly reflecting that an interdisciplinary tumor board with dedicated experienced neuroradiologists and nuclear medicine physicians had put forward the biopsy indications. Most previously published studies comprised sample sizes of a few dozen to a couple hundred patients (28–36). The largest retrospective monocentric study comprised 622 patients biopsied over the course of 20 years as compared to a sample size of 1,214 patients over 5 years reported in our study (28, 30). The rate of biopsies investigating suspected tumor recurrence is relatively high, as we provide an effective, low-risk stereotactic biopsy technique and have many patients with suspected recurrences coming to our tertiary referral center for second opinions and to get a tissue-based diagnosis, which is decisive to maintain a successful therapy or enable an informed change of therapy. Unspecific therapy-related changes and pseudoprogression phenomena mimicking tumor relapse gain more importance in light of emerging immunotherapies (37). In our series, more than one in ten (12.5%) of suspected tumor recurrences showed only therapy-induced changes histologically, obviating the need for more invasive procedures in this patient collective. In addition, in analogy to solid cancers and brain metastases, the search for druggable targets in newly diagnosed and recurrent gliomas just embarks and will increase in the future. As new therapies being recommended by a molecular tumor board become available, tissue diagnosis of possible druggable targets should not be withheld from “biopsy-only” patients. Consequently, in all cases where open microsurgical resection is not deemed feasible or medically justified and in all “diagnostic-only” situations, the need for a minimal invasive and maximal effective technique to obtain an informative diagnostic material is beyond doubt. This has also been adopted now for diffuse brainstem gliomas (38, 39).

Earlier, small biopsies did not yield enough viable tissue for obtaining a valid and, presently, mandatory molecular diagnosis; however, the contemporary refined technologies of molecular biology enable the analysis of a panel of different molecular markers even from very small specimens (40, 41). Only with access to elaborate the neuropathological technique and expertise, stereotactic biopsies are adequate to gain all diagnostic information in case open resection is not deemed feasible or justified. In our series, over 96% of biopsies were informative concerning histology and the molecular signature of the tumor. Prerequisite for a proper molecular diagnosis is to obtain the material out of the solid parts of the tumor since any “contamination” of the specimen with either normal adjacent brain or else tumor necrosis might hamper diagnostic yield and accuracy. Moreover, the neuropathologist has to be experienced in working up these small samples. In our practice, the pathologist is on site in the OR during the procedure to check *via* smear preparation whether the material obtained is sufficient in terms of quantity and quality for diagnosis.

Serial sampling with multiple specimens along the trajectory allows to “map” the tumor, including its infiltration zone. This is extremely useful in heterogeneously composed tumors where one single biopsy might lead to a sampling error like misdiagnosing or undiagnosed. MR features such as contrast enhancement on T1-weighted imaging or cell density on T2-weighted sequences can highlight the suspicious areas that should be targeted preferentially. PET with amino acid tracers such as [^18^F]FET, [^11^C]Methionine, or [^18^F]FDOPA are particularly useful to detect the relevant areas for diagnostic biopsies in either diffuse, non-contrast enhancing gliomas or in multimodally pretreated lesions with differential diagnosis of recurrent tumor vs. treatment-related phenomena (12, 13, 42, 43). While [^18^F]FET PET and perfusion MRI can give important hints about the likelihood of true progression vs. pseudoprogression (12), our data support the continued use of histology as the gold standard for identifying both with high reliability and low risk. Furthermore, image-guided biopsies allow to precisely target and sample different areas within heterogeneously composed tumors to address the mutational and clonal analyses with a high spatial resolution.

As long as molecular alterations within the tumor are homogeneously distributed, sampling errors are not an issue. Referring to this, the homogeneous distribution of the alteration has to be shown in a systemical order to elucidate whether a risk of sampling error might be relevant for a given particular marker. This has been demonstrated for most of the relevant basic molecular signatures in gliomas (26, 43, 44). The earlier a molecular alteration appears in the timeline of tumor evolution, the more likely it can appear homogeneously within the tissue (45). Conversely, especially for late events, more heterogeneous patterns evolve, which have to be taken into account for biopsy (46).

The patterns of either diagnostic or therapeutic targets may change during the course of disease, so recurrent tumors may have a completely different pattern compared to the original newly diagnosed tumor. Again, early events in the tumorigenesis may not change, whereas new subclones during tumor progression may carry new mutations (45). Especially, therapy-driven alterations and an increase in mutational burden may necessitate re-biopsy (47–50). Whereas, *MGMT* promoter methylation does not change over time (51), other therapy relevant markers do (52, 53). Hence, it may not justified to include patients with recurrent tumors into clinical trials for targeted therapy just on the basis of the initial specimen. Instead, dependent on the target, the molecular status has to be newly defined by either resection or biopsy (54, 55).

The complication rate was low with only 0.6% permanent and 0.6% transient severe complications overall. In the subgroup of brainstem lesions, moderate or severe complications occurred at a slightly higher rate of 2.6%. Thus, even in patients with gliomas located in delicate areas such as the brainstem or the midbrain, tissue can be acquired with a low risk of permanent deficit and a high diagnostic yield. The low complication rate reported in this study justifies the application of stereotactic biopsies less reluctantly whenever diagnostic uncertainties occur during the course of disease and treatment. The low number of symptomatic hemorrhages suggests waiving the routine CT scan. Previous series of frame-based biopsies report mortality rates of 0.7–4% (28, 30–36). Post-procedural morbidity (i.e., transient or permanent neurological deficits, epileptic seizures, coma) ranged from 3 to 13%. Asymptomatic bleedings on postoperative CT scans have been reported in up to 60% of patients and symptomatic bleedings occurred in up to 8.6% of cases. In our series with no mortality, the rate of severe transient and permanent complications was much lower. In previous studies, brain biopsies typically yielded diagnoses at rates of 89–92% and even higher when intraoperative histological smears were carried out (21, 28, 31, 56–59). By comparing frame-based with frameless biopsies, no clear advantage of either technique regarding complication rates or diagnostic yield could be shown so far (29, 32, 57–60). In our experience, a high personal and interdisciplinary expertise is required to obtain constant procedural safety and efficiency. A high caseload being taken care of by a group of few dedicated neurosurgeons is, in our opinion, important. In addition, high-resolution vascular imaging, including MR and CT angiography, meticulous planning of the trajectories by avoiding vessels, ventricular puncture, and arachnoidal contact, as the subarachnoid space is especially prone to hemorrhage, is required. Furthermore, the presence of a dedicated neuropathologist on site not only ensures specimen quality but also prevents an unnecessary high number of specimens, which is especially important in delicate locations. Also, as always in neurosurgery, proper selection of indications and patients is key. Despite low complication rates, the indication for brain biopsy must be strict as it still is an invasive procedure.

In the future, determination of changes in the molecular signature of gliomas and very early detection of therapy response or failure will gain further importance. Whether several techniques and concepts of “liquid biopsy” using CSF, plasma, or even urine may complement or even replace stereotactic biopsies for at least some indications remains yet uncertain (61–66). Also, molecular imaging using novel specific tracers might help to non-invasively better characterize gliomas in the future (67, 68).

With a mean duration of 50 min, frame-based biopsy in a streamlined setting is a time- and cost-efficient procedure. At our institution, we can perform up to five biopsies in the same OR within the regular working hours. We could obtain a high diagnostic yield with a very low rate of either inconclusive biopsies or complications. This leads to a low rate of re-biopsies, which is an important factor for both the safety and the effectiveness in the process of decision making and patient management. Hence, we consider the balance between the complexity and the costs on one side and the benefit for the patient/patient management on the other side to be in due proportion.

## Conclusion

In conclusion, a streamlined stereotactic biopsy procedure proved to be time-effective and low-risk in primary and recurrent glioma. A high diagnostic yield enables the diagnostics of molecular markers, as required by the current WHO classification, as well as in the increasingly important context of molecular tumor boards. A postoperative CT scan should only be performed when clinically indicated. A good technical setup with easily accessible CT and a specialized team for trajectory planning and neuropathological analysis are recommended.

## Data Availability Statement

The datasets presented in this article are not readily available because of national and institutional laws to protect patient confidentiality. Requests to access the datasets should be directed to the Center for Neuropathology and Prion Research of the University Hospital of Munich.

## Author Contributions

JT and SQ contributed to the conception and design of the study. AD, SK, and SQ organized the database, evaluated the clinical courses, and performed the image analyses. SQ carried out the statistical analysis. SK, SQ, JW, and JT wrote the manuscript. All authors contributed to the manuscript revision, read, and approved the submitted version.

## Funding

This project was partly funded by the Deutsche Forschungsgemeinschaft (DFG, German Research Foundation) (FOR 2858 Project Number 421887978).

## Conflict of Interest

The authors declare that the research was conducted in the absence of any commercial or financial relationships that could be construed as a potential conflict of interest.

## Publisher's Note

All claims expressed in this article are solely those of the authors and do not necessarily represent those of their affiliated organizations, or those of the publisher, the editors and the reviewers. Any product that may be evaluated in this article, or claim that may be made by its manufacturer, is not guaranteed or endorsed by the publisher.
