# Supplementary material for: Diagnostic Yield and Complication Rate of Stereotactic Biopsies in Precision Medicine of Gliomas
Source: Front Neurol. 2022 Mar 30;13:822362. doi: 10.3389/fneur.2022.822362 (PMC9005817; doi:10.3389/fneur.2022.822362)
Supplement: Supplementary file 1 [file Table_1.PDF]

An Adverse Event (AE) is any unfavorable and unintended sign (including an abnormal laboratory finding), symptom, or disease temporally associated with the use of a medical treatment or procedure that may or may not be considered related to the medical treatment or procedure. An AE is a term that is a unique representation of a specific event used for medical documentation and scientific analyses.	
Grade 1	Mild; asymptomatic or mild symptoms; clinical or diagnostic observations only; intervention not indicated.
Grade 2	Moderate; minimal, local or noninvasive intervention indicated; limiting age-appropriate instrumental ADL*.
Grade 3	Severe or medically significant but not immediately life-threatening; hospitalization or prolongation of hospitalization indicated; disabling; limiting self care ADL**.
Grade 4	Life-threatening consequences; urgent intervention indicated.
Grade 5	Death related to AE.
*Instrumental Activities of Daily Living (ADL) refer to preparing meals, shopping for groceries or clothes, using the telephone, managing money, etc.<br>**Self care ADL refer to bathing, dressing and undressing, feeding self, using the toilet, taking medications, and not bedridden.	
